# Lamin-A interacting protein Hsp90 is required for DNA damage repair and chemoresistance of ovarian cancer cells

**DOI:** 10.1038/s41419-021-04074-z

**Published:** 2021-08-12

**Authors:** Yixuan Wang, Quan Chen, Di Wu, Qifeng Chen, Guanghui Gong, Liuqing He, Xiaoying Wu

**Affiliations:** 1 Department of Pathology, Xiangya Hospital, Central South University, Changsha, 410008 Hunan Province P. R. China; 2 Department of Pathology, School of Basic Medical Science, Central South University, Changsha, 410008 Hunan Province P. R. China

**Keywords:** Cancer therapy, Gynaecological cancer, Gynaecological cancer

## Abstract

Ovarian cancer is the most malignant gynecologic cancer. Previous studies found that lamin-A was associated with DNA damage repair proteins but the underlying mechanism remains unclear. We speculate that this may be related to its interacting proteins, such as Hsp90. The aim of this study is to investigate the effects of Hsp90 on DNA damage repair and chemoresistance of ovarian cancer cells. In our research, co-immunoprecipitation (co-IP) and mass spectrometry (MS) were used to identify proteins interacting with lamin-A and the interaction domain. Next, the relationship between lamin-A and Hsp90 was explored by Western blotting (WB) and immunofluorescence staining. Then, effect of Hsp90 inhibition on DNA damage repair was assessed through detecting Rad50 and Ku80 by WB. Furthermore, to test the roles of 17-AAG on cell chemosensitivity, CCK-8 and colony formation assay were carried out. Meanwhile, IC50 of cells were calculated, followed by immunofluorescence to detect DNA damage. At last, the mouse xenograft model was used in determining the capacity of 17-AAG and DDP to suppress tumor growth and metastatic potential. The results showed that lamin-A could interact with Hsp90 via the domain of lamin-A^1-430^. Besides, the distribution of Hsp90 could be affected by lamin-A. After lamin-A knockdown, Hsp90 decreased in the cytoplasm and increased in the nucleus, suggesting that the interaction between lamin-A and Hsp90 may be related to the nucleocytoplasmic transport of Hsp90. Moreover, inhibition of Hsp90 led to an obvious decrease in the expression of DSBs (DNA double-strand break) repair proteins, as well as cell proliferation ability upon DDP treatment and IC50 of DDP, causing more serious DNA damage. In addition, the combination of 17-AAG and DDP restrained the growth of ovarian cancer efficiently in vivo and prolonged the survival time of tumor-bearing mice.

## Introduction

Lamin-A is the major components of the nuclear lamina [1]. Nuclear lamina locates below the INM (inner nuclear membrane), and is required in maintaining nuclear structure and integrity [2]. Lamin-A is encoded by the LMNA gene, which plays an important role in a series of cell processes, such as regulation of gene expression, senescence, chromatin organization, and DNA repair [3, 4]. Many studies demonstrated that mutations in LMNA or defects of lamin-A were related to laminopathy, such as HGPS (Hutchinson–Gilford Progeria Syndrome). Laminopathy was associated with increased genomic instability [5]. Fibroblasts from HGPS patients showed increased levels of DSBs, increased chromosomal instability, and defects of DSBs repair [6]. Similar findings were found in many studies, supporting that lamin-A played an important role in regulating DNA damage repair [7–9]. However, the underlying mechanism of lamin-A in DNA repair remains to be further studied.

Lamin consists of a NLS (nuclear localization sequence) and an Ig-like domain [10]. This unique structure enables lamin not only to form a complex meshwork of proteins that providing mechanical support to maintain nuclear stability but also to play an important role as a signal molecule through interacting with other proteins [5, 11, 12]. Previous studies demonstrated that ovarian cancer cells that migrated through restricted pores decreased after further knockdown of lamin-A, and the expressions of DSBs repair proteins were significantly downregulated [13]. We suspect that this may be related to the interaction proteins of lamin-A, such as Hsp90.

Hsp90 is a highly conserved chaperone, which controls the stability and activity of chaperone proteins [14] and maintains the functions of proteins in tumors. It was found that Hsp90 increased in breast cancer and was associated with a poor survival rate [15]. Some studies showed that Hsp90 was related to tumor cell transformation and invasion [16] but its role in ovarian cancer has not been fully studied.

Hsp90 can stabilize and activate more than 200 proteins [17–19], and can interact with other proteins to help them obtain active forms, involving in protein homeostasis, chromatin remodeling, and DNA repair [20]. Hsp90 can interact with about 10% eukaryotic proteins [21], and over 725 interactions have been confirmed. This allows Hsp90 to function as a network hub linking diverse protein interaction networks [22]. Among the Hsp90 clients identified, there are many proteins involved in DNA damage repair, such as DSBs repair MRN complex and SSBs (DNA single-strand break) repair CHK1. In addition, inhibition of Hsp90 can lead to DNA repair defects (such as homologous recombination repair [23, 24]), enhance the cytotoxicity of DNA damaging agents [25–27], making cells more sensitive to DNA damage [28, 29]. 17-AAG (Allylaminogeldanamycin), an inhibitor of Hsp90, has been used in clinical trials of breast cancer and other cancers [30]. All the findings indicate that Hsp90 inhibitors could play an important role in inhibiting tumor growth, reducing metastasis potential, and increasing tumor sensitivity to chemoradiotherapy.

However, the specific role of Hsp90 in DNA damage and repair of ovarian cancer cells remains unclear. As the most malignant gynecologic cancer, ovarian cancer is prone to invasion, metastasis, recurrence, and is easy to develop drug resistance. In this study, the effects of lamin-A interacting protein Hsp90 on DNA damage and drug resistance of ovarian cancer cells were investigated, so as to find a new direction for the treatment of ovarian cancer.

## Materials and methods

### Cell lines and cell culture

293 T (HEK) cells and ovarian cancer cell lines HO-8910, SKOV-3 were purchased from CCTCC (China Center for Type Culture Collection, Wuhan, China). SKOV-3ip cell line is a highly metastatic cell line derived from SKOV-3. SKOV-3ip cells were kindly donated by Professor Xin Lu (Obstetrics and Gynecology Hospital, Fudan University, Shanghai, China). Cells were cultured using standard protocols. Two hundred and ninety-three T cells were cultured in DMEM high-glucose media (BI, Biological Industries, Israel), HO-8910, SKOV-3, and SKOV-3ip cells were cultured in RPMI-1640 media (BI), supplemented with 10% fetal bovine serum (BI). All cells were maintained at 37 °C under 5% CO_2_.

### Lamin-A overexpression

The plasmids encoding lamin-A with flag tag were purchased from GENECHEM (Shanghai, China). Cells were transfected with flag-lamin-A and the corresponding empty vector. Before transfection, cells were plated at 2 × 10^5^ cells/well in 6-well cell culture plates. Then 2 μg plasmid and 4 μL lipofectamine2000 (Life Technologies, Carlsbad, CA, USA) were diluted in 0.2 mL Opti-MEM medium (GIBCO, Carlsbad, CA, USA) following the manufacturer’s instructions. Subsequently, cells were maintained at 37 °C for 6–8 h, then the mixture was replaced by 2 mL of RPMI-1640 complete culture medium. Western blotting and qRT-PCR were performed after 24 h to detect the overexpression efficiency.

### Lamin-A knockdown

All GFP-shRNAs were purchased from GENECHEM. Before transfection, HO-8910 cells were plated at 2 × 10^5^ cells/ well in 6-well cell culture plates. The transfection complex was prepared in Opti-MEM reduced serum medium following the manufacturer’s instructions. A complex of 2 μg shRNA and 4 μL Lipofectamine2000 (1 μg/mL) was prepared. A single dose was denoted as “shRNA + ” to achieve moderate knockdown, and three doses to achieve further knockdown were denoted as “shRNA + ++”, compared with those using the same doses of scrambled shRNA (scr shRNA) in transient knockdown cells. Then, cells were maintained at 37 °C for 6–8 h, and the medium was replaced with RPMI-1640 complete medium. For transient transfection, knockdown efficiency was determined by Western blotting, qRT-PCR, and immunofluorescence staining after 24 h. For stable knockdown, cells were plated into 6-well cell culture plates with selection medium (1 mg/mL G418) at 1, 1:10, 1:100, and 1:1000 dilutions. G418 was maintained for 10 days to obtain shRNA stably expressing cells. Then, single colonies were visible and were picked with a pipet tip, followed by plating in 24-well plates. Cells were transferred to appropriate plates when the confluence reached 80–90%. Liquid nitrogen was prepared and cells were stored for subsequent experiments.

### Cytoplasmic and nuclear protein fractionation

Nuclear and cytoplasmic extracts were obtained using NE-PER kit (P0027, Beyotime Biotechnology, China). Cells were washed with PBS (phosphate-buffered saline), scraped off, and collected by centrifugation. Then, 200 μL of cytoplasmic protein extraction reagent A was added to every 20 μL of cell precipitation. Next, the tube was vortexed for 5 s to completely suspend cell precipitate and placed on ice for 10–15 min. Then, 10 μL of cytoplasmic protein extraction reagent B was added, followed by vortex agitation for 5 s. After centrifugation at 4 °C (12000–16000 g, 5 min), the supernatant was immediately collected into a precooled plastic tube, which was the extracted cytoplasmic protein. Next, 50 μL of nucleoprotein extraction reagent was added into the precipitation, followed by vortex agitation for 15–30 s. Subsequently, the mixture was put on ice and vortexed 15–30 s every 1–2 min for 30 min. Finally, after centrifugation at 4 °C for 10 min (12000–16000g), the supernatant was collected immediately into a precooled plastic tube, which was the extracted nuclear protein.

### Western blotting

Cells were lysed in lysis buffer containing protease inhibitor. Protein concentration was determined by the BCA protein assay kit (Novagen, Merck Group, Madison, USA). Equal amounts of denatured proteins were separated by SDS-PAGE gels and then transferred onto PVDF membranes (Millipore, Bedford, MA, USA). The membranes were blocked in 5% nonfat milk at room temperature for 1 h, and then incubated with primary antibody, followed by horseradish peroxidase-conjugated secondary antibody. Protein expression levels were detected using Image Lab software (Bio-Rad, CA, USA). Antibodies against lamin-A (mouse, ab-8980) and γ-H2AX (rabbit, ab81299) were from Abcam, U.K. Antibodies against Rad50 (rabbit, 3427) and Ku80 (rabbit, 2180) were from Cell Signaling Technology, USA. Antibody against Flag (mouse, F1804) was from sigma, USA. Antibodies against His (mouse, 66005–1-Ig) and Hsp90 (rabbit, 13171-1-AP) were from Proteintech, China.

### Co-IP

Cells (approximately 2–5 × 10^7^) were washed with PBS and lysed in lysis buffer (Cell lysis buffer for Western and IP, P0013, Beyotime Biotechnology, China) containing protease inhibitor for 10 min on ice. After centrifugation, the supernatant was collected and precleared with protein G PLUS-Agarose beads (Santa Cruz, USA) at 4 °C for 60 min. Protein concentrations were normalized by BCA protein assay kit, and the precleared lysate was then incubated with flag or his antibody, as well as protein G PLUS-Agarose beads overnight at 4 °C. Immunoprecipitates were collected by centrifugation and washed three times with PBS. Then the beads complexed with the immunoprecipitated proteins were resuspended in SDS loading buffer and boiled at 100 °C for 5 min, processed for Western blotting analysis. 2.5% of whole cell lysate was taken as input for each immunoprecipitation.

### Mass spectrometry (MS) analysis

The protein lysates were separated by SDS-PAGE. The gels were stained with coomassie brilliant blue for 45 min and then washed three times. The SDS-PAGE bands were cut off, collected into eppendorf tube, and washed in ultrapure water for three times. Then, decolorizing solution (25% ACN, 25 mM NH_4_HCO_3_) was added into the tube, followed by dehydrated with ACN, and dried in a vacuum for 30 min. Next, the reducing solution (25 mM DTT, 25 mM NH_4_HCO_3_) was added in the tube and reacted at 56 °C for 30–60 min. The protein was dehydrated again after alkylation by adding a protein protection solution (50 mM IAA, 25 mM NH_4_HCO_3_). Trypsin was used for enzymolysis. 50%ACN and 0.1% FA (trifluoroacetic acid) were added. Finally, the peptide was extracted and analyzed by mass spectrometry with an LTQ Orbitrap XL (Thermo Fisher Scientific, Waltham, MA, USA) according to the manufacturer’s instructions.

### Immunofluorescence staining

Glass slides were fixed in 4% paraformaldehyde for 30 min at room temperature followed by PBS washing. Then cells were permeabilized by 0.5% TritonX-100 for 20 min, blocked by 5% BSA, and incubated in the primary antibody at room temperature for 30 min, and stayed overnight at 4 °C. Finally, the primary antibody was tagged with the corresponding secondary antibody for 1 h at room temperature. Images were obtained with Olympus DP73 fluorescence microscope.

### Colony formation assay

Cells were seeded into 6-well plates in triplicates at a density of 1000 cells/well. Cells were treated with cisplatin (DDP, 2.5 μM, Sigma-Aldrich, St. Louis, MO, USA), or 17-AAG (0.5 μM, APExBIO Technology, Houston, TX, USA). After 14 days, cells were washed three times with PBS, fixed in 4% paraformaldehyde for 10 min, and then stained with 0.1% crystal violet for 20 min at room temperature. Colonies containing more than 50 cells were counted.

### Drug sensitivity assay

The drug sensitivity assay was performed using Cell Counting Kit-8 (CCK-8, Dojindo, Japan) according to the manufacturer’s instructions. Cells were plated in 96-well plates. To determine the individual IC50 of DDP, rising drug concentrations (2.5, 5, 10, 20, or 40 μM) were applied for 96 h and relative cell viability was assessed by luminometry. Vehicle-treated control cells were set to 100% cell viability and the relative cell viability on drug treatment was calculated from three independent biological replicates for each drug concentration. IC50 was calculated by Graphpad Prism 5.0.

### Animal model studies

All animal experiments were conducted according to the protocols approved by the Animal Care and Use Committee of Central South University, China. The mice (female 4–5weeks old, 18–20 g BALB/c nude mice) were randomly assigned to three groups (*n* = 4/group). The luciferase-expressing HO-8910 cells (1 × 10^6^ cells) were injected intraperitoneally into three groups. After two weeks, mice were intraperitoneally injected with DDP (3 mg/kg), DDP combined with 17-AAG (50 mg/kg), or the same amount of normal saline as control once a week for up to 6 weeks. For bioluminescence imaging of living animals, mice were injected with 100 mg/kg D-luciferin (Caliper Life Sciences, Hopkinton, MA, USA) in PBS, then anesthetized with 2.0% isofluorane, and imaged by IVIS system (Bruker, Billerica, MA, USA). Bioluminescence images were acquired with a Bruker MI SE imaging system.

### Statistical analysis

All data were presented as the mean values ± the standard error of the mean from at least three independent experiments. Two-tailed Student’s *t* test was used for comparison between two groups. Statistical analyses were carried out using Graphpad Prism 5.0. The error bars indicate the standard deviation in all the figures. *p* value < 0.05 was considered statistically significant. *p < 0.05, **p < 0.01, ***p < 0.001.

## Results

### Identification of lamin-A interacting proteins by MS

**Fig. 1:**
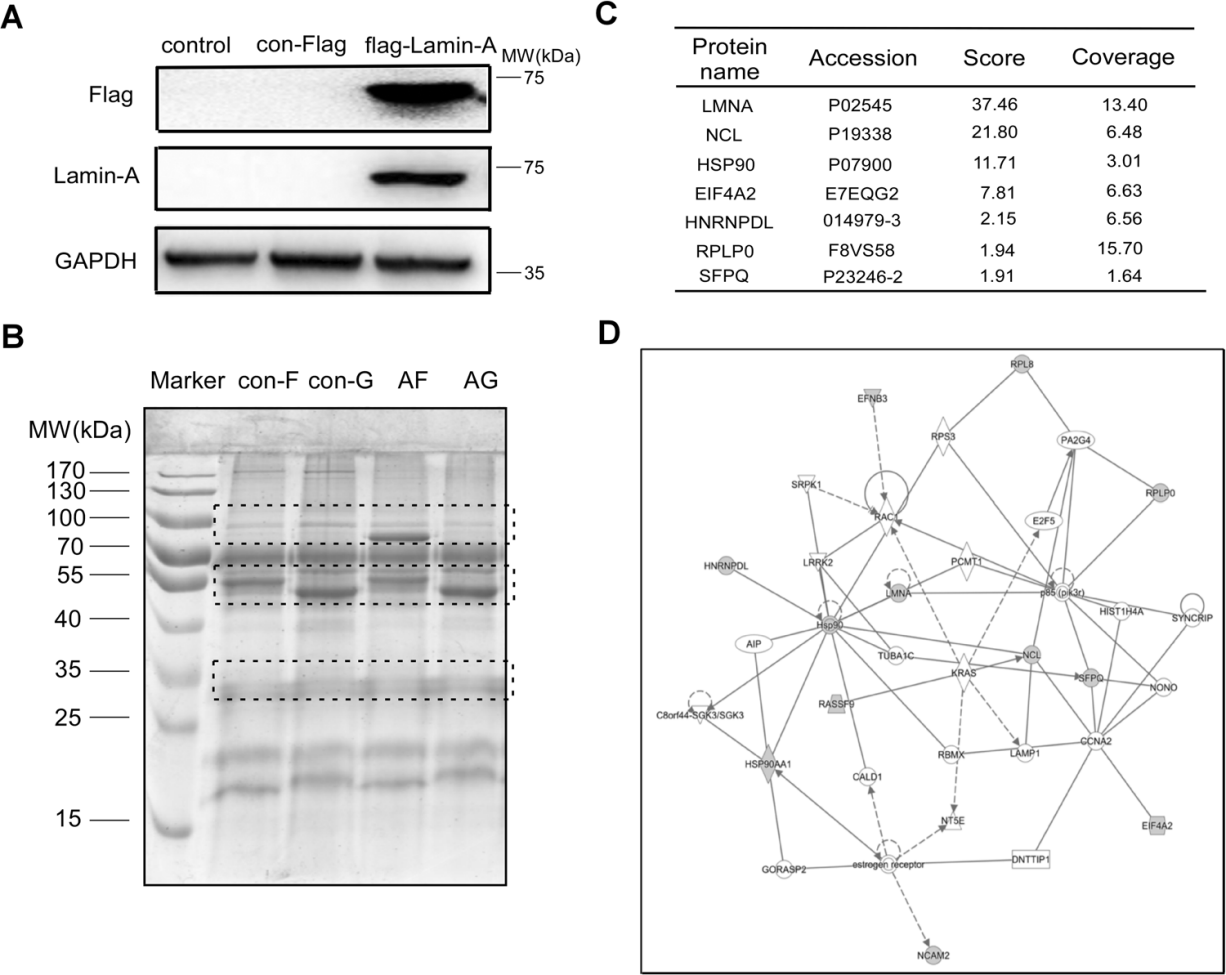
Identification of lamin-A interacting proteins by MS. **A** Flag-lamin-A or con-flag was transfected into 293 T cells. The control was 293 T cells without transfection, and the con-flag was 293 T cells transfected with empty flag vector. Lamin-A level was detected by Western blotting, GAPDH was used as the endogenous reference protein. **B** 293 T cells were transfected with flag-lamin-A or con-flag plasmid and the cell lysates were co-immunoprecipitated by flag antibody or IgG antibody as control. Differential bands were shown in Coomassie staining SDS-PAGE gels. Con-F: flag antibody and 293 T cells transfected with con-flag, con-G: IgG antibody and 293 T cells transfected with con-flag, AF: flag antibody and 293 T cells transfected with flag-lamin-A, AG: IgG antibody and 293 T cells transfected with flag-lamin-A. **C** Top candidates of lamin-A interacting proteins identified by co-IP and MS analysis. Score: Protein scores, calculated by Proteome Discoverer application from a list of peptides, indicate the relevance of a protein. Coverage: Coverage of identified high-confidence peptides match the protein. **D** Network diagram of lamin-A interacting proteins by IPA.

In our previous studies, lamin-A was found to be related to the migration and DNA damage repair of ovarian cancer cells [13] but the underlying molecular mechanisms were poorly understood. We suspect that lamin-A may affect this process through its interacting proteins. For further study, proteins interacted with lamin-A were analyzed. Firstly, the flag-lamin-A vector was constructed and transfected into 293 T cells, and the expression was detected by Western blotting, as shown in Fig. 1A. Next, 293 T cells were transfected with the flag-lamin-A plasmid and the cell lysates were co-immunoprecipitated by Protein G Plus-Agarose and flag antibody, followed by SDS-PAGE (Fig. 1B). After Coomassie blue staining, the bands were excised from gel (marked with dotted dashed box) and detected by MS, and a series of proteins that might interact with lamin-A were identified. Then the network diagram was obtained by IPA software (Fig. 1D). Seven of the proteins had a score of at least 1.91, and LMNA was ranked number 1 among them, with a score of 37.46 (Fig. 1C). The protein ranked third on the list was Hsp90, a highly and unique chaperone with a wide range of functions, and was found to be related to DNA damage and repair [31].

## Lamin-A can interact with Hsp90 via the domain of lamin-A^1-430^

**Fig. 2:**
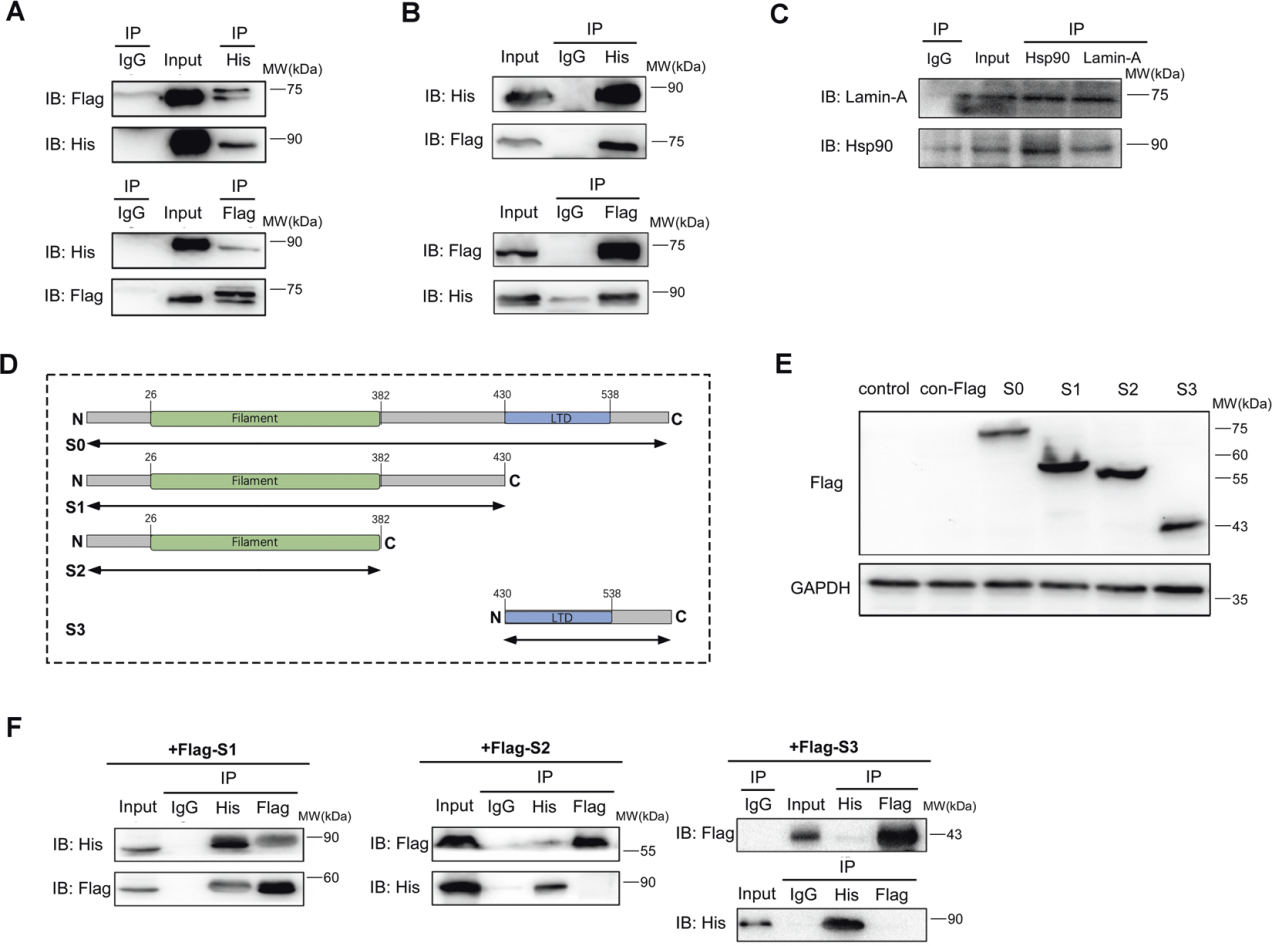
Lamin-A can interact with Hsp90 via the domain of lamin-A^1-430^. **A** and **B** Flag-tagged lamin-A (flag-lamin-A) and his-tagged Hsp90 (his-Hsp90) were co-expressed in cells for co-IP analysis using antiflag or antihis antibodies (A: HO-8910 cells, B: 293 T cells). Flag-lamin-A and his-Hsp90 were detected with their specific antibodies, respectively. Co-IP with IgG serves as a negative control. **C** Lamin-A and Hsp90 could interact in HO-8910 cells without transfecting the exogenous tagged plasmid. **D** Domains of lamin-A and its different derivatives (S0–S3) constructed. **E** The expression of truncations in 293 T cells, with GAPDH as the internal reference. **F** His-Hsp90 and truncations of lamin-A (S1–S3) were co-expressed in 293 T cells for co-IP analysis using antiflag or antihis antibodies. Flag-lamin-A and his-Hsp90 were detected with their specific antibodies, respectively. Co-IP with IgG serves as a negative control.

Co-IP was carried out to verify the interaction between lamin-A and Hsp90. Flag-lamin-A and his-Hsp90 were co-transfected into 293 T cells and HO-8910 cells, respectively. As shown in Figs. 2A and 2B, his-Hsp90 could be detected in the complexes precipitated by flag antibody in HO-8910 cells and 293 T cells. At the same time, flag-lamin-A could also be detected in the complexes precipitated by his antibody, indicating that there was an interaction between lamin-A and Hsp90. Besides, we also discovered the interaction between them in HO-8910 cells without transfecting the exogenous tagged plasmid and got similar results (Fig. 2C), further confirming that lamin-A and Hsp90 could interact with each other in cells.

To determine the interaction domain, plasmids expressing different flag-tagged lamin-A derivatives were constructed, namely the full-length WT lamin-A^1-664^ (S0), lamin-A^1-430^ (S1), lamin-A^1-382^ (S2), and lamin-A^430-664^ (S3) (Fig. 2D). Then, plasmids were transfected into 293 T cells and confirmed by Western blotting (Fig. 2E).

Furthermore, these flag-lamin-A-derived plasmids were co-transfected with his-tagged Hsp90 into 293 T cells. Co-IP analysis showed that, except for S2 and S3, the full-length WT lamin-A and truncated lamin-A S1 successfully pulled down his-Hsp90 (Fig. 2F), suggesting that lamin-A^1-430^ region served as the domain for interaction with Hsp90.

## Knockdown of lamin-A affects the distribution of Hsp90 in cells

**Fig. 3:**
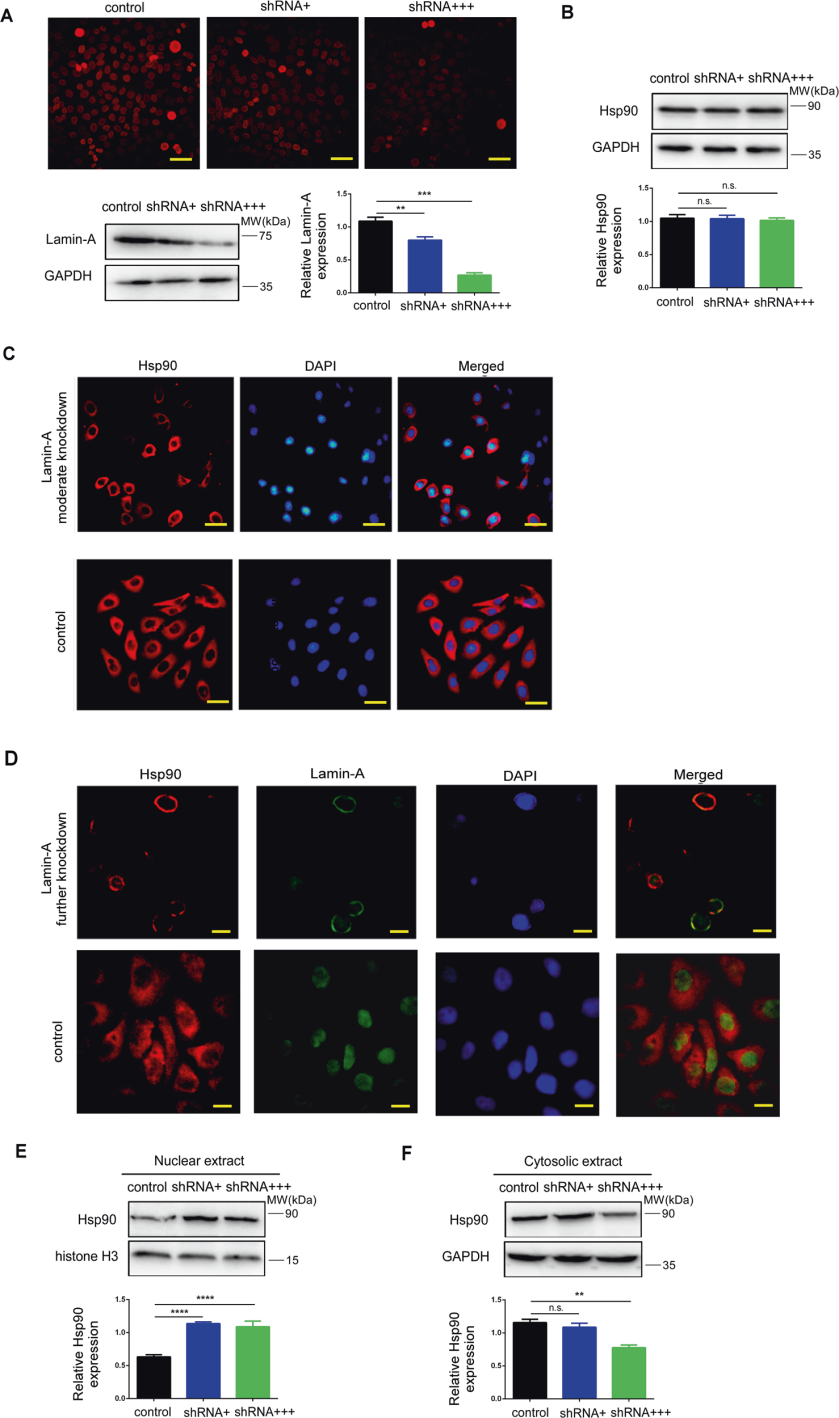
Knockdown of lamin-A affects the distribution of Hsp90 in cells. **A** Representative images of cells stained for lamin-A, as well as the protein levels and quantitative results of lamin-A measured by Western blotting, GAPDH was used as the endogenous reference. HO-8910 cells were transfected with shRNA + , shRNA + ++, and scrambled vector as control. Scale bars, 50 μm. **B** The protein levels of Hsp90 were measured by Western blotting, HO-8910 cells were transfected with shRNA + , shRNA + ++, and scrambled vector as control, GAPDH was used as the endogenous reference protein. **C** Representative images of cells stained for Hsp90 (red) and DNA (DAPI, blue). HO-8910 cells were transfected with shRNA + and scrambled vector as control. Scale bars, 50 μm. **D** Representative images of cells stained for Hsp90 (red), lamin-A (green), and DNA (DAPI, blue). HO-8910 cells were transfected with shRNA + ++ and scrambled vector as control. Scale bars, 25 μm. E: The protein levels of Hsp90 in the nucleus were measured by Western blotting after lamin-A moderate and further knockdown, histone H3 was used as the endogenous reference protein. **F** The protein levels of Hsp90 in the cytoplasm were measured by Western blotting after lamin-A moderate and further knockdown, GAPDH was used as the endogenous reference protein. All the experiments were repeated three times. All the error bars indicated means ± SD. Statistical significance was concluded at *p < 0.05; **p < 0.01; ***p < 0.001.

Next, the relationship between lamin-A and Hsp90 was further explored. Transient transfected HO-8910 cells with shRNA to moderate knockdown or further knockdown lamin-A, as previously described [13]. Knockdown efficiency was detected by Western blotting and IF (Fig. 3A). Total proteins were extracted 24 h after transfection. Then Western blotting was used to detect the expression of Hsp90. As shown in Fig. 3B, the level of Hsp90 did not change significantly.

Then, we suspect that if the distribution of Hsp90 could be influenced by lamin-A, thus affecting DNA damage repair proteins. For further confirmation, lamin-A was a moderate knockdown in HO-8910 cells. Immunofluorescence staining indicated that the expression of Hsp90 in the cytoplasm decreased (Fig. 3C), suggesting that lamin-A could lead to the redistribution of Hsp90. In addition, we noticed that in the further knockdown group, lamin-A and Hsp90 predominantly co-localized around the nuclear membrane (Fig. 3D).

To further investigate the distribution of Hsp90, Hsp90 levels in the nucleus and cytoplasm were measured after the separation of nucleoplasmic proteins. Western blotting showed that Hsp90 decreased in the cytoplasm after lamin-A knockdown, and accumulated in the nucleus compared with the untreated control cells (Figs. 3E, 3F), proving that lamin-A could cause the redistribution of Hsp90.

## Inhibition of Hsp90 reduces expression of DSBs repair proteins

**Fig. 4:**
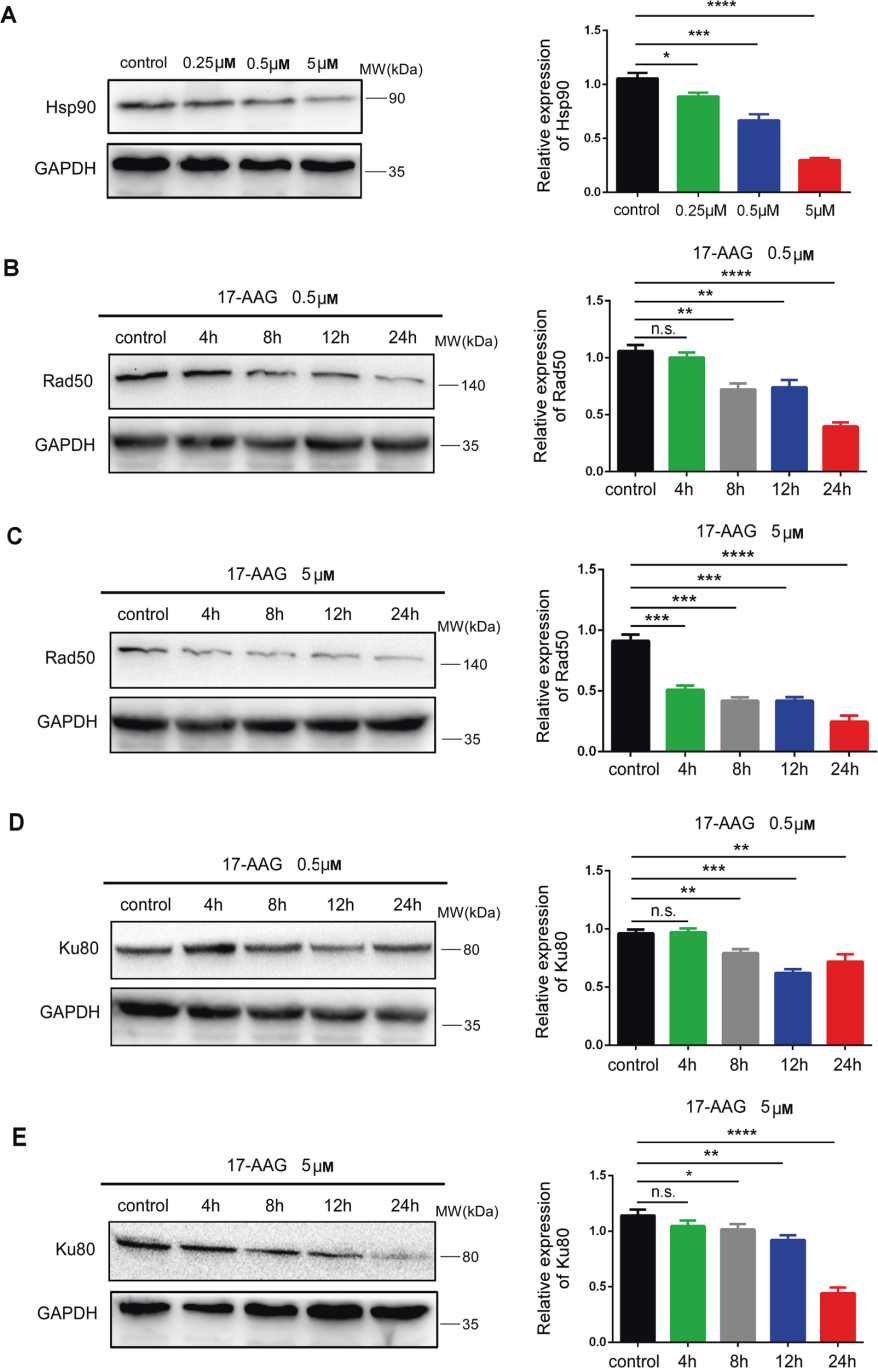
Inhibition of Hsp90 reduces expression of DSBs repair proteins. **A** The protein levels of Hsp90 were measured by Western blotting in HO-8910 cells treated with or without 17-AAG (0.25, 0.5, and 5 μM), GAPDH was used as the endogenous reference protein. **B** The protein levels of Rad50 were measured at 4, 8, 12, and 24 h by Western blotting in HO-8910 cells treated with or without 0.5 μM 17-AAG, GAPDH was used as the endogenous reference protein. **C** The protein levels of Rad50 were measured at 4, 8, 12, and 24 h by Western blotting in HO-8910 cells treated with or without 5 μM 17-AAG, GAPDH was used as the endogenous reference protein. **D** The protein levels of Ku80 were measured at 4, 8, 12, and 24 h by Western blotting in HO-8910 cells treated with or without 0.5 μM 17-AAG, GAPDH was used as the endogenous reference protein. **E** The protein levels of Ku80 were measured at 4, 8, 12, and 24 h by Western blotting in HO-8910 cells treated with or without 5 μM 17-AAG, GAPDH was used as the endogenous reference protein. All the experiments were repeated three times. All the error bars indicated means ± SD. Statistical significance was concluded at *p < 0.05; **p < 0.01; ***p < 0.001.

Rad50 and Ku80 are important proteins in the DSBs repair process. In order to further study the effect of Hsp90 inhibition on DNA damage and repair, Rad50 and Ku80 were detected. HO-8910 cells were treated with different concentrations of Hsp90 inhibitor 17-AAG, and cells treated without 17-AAG were regarded as the control group. Western blotting was used to detect Hsp90 expression, respectively. As showed in Fig. 4A, Hsp90 was significantly downregulated.

Then, proteins were extracted at different time points (4, 8, 12, and 24 h) to detect the expression of Rad50 and Ku80, which were proved to be downregulated after further knockdown of lamin-A [13]. Results showed that the levels of Rad50 and Ku80 decreased after 8 h in cells treated with relative low concentration of 17-AAG (0.5 μM), as shown in Figs. 4B and 4D. In the groups treated with relative high concentration of 17-AAG (5 μM), Rad50 significantly decreased after 4 h (Fig. 4C), and Ku80 also decreased after 8 h (Fig. 4E), indicating that DSBs repair proteins were downregulated in a dose- and time-depend manner and Hsp90 inhibition could affect DSBs repair, which was similar to lamin-A deficiency [13].

## Combination of 17-AAG and DDP can efficiently inhibit the growth of ovarian cancer cells in vitro

**Fig. 5:**
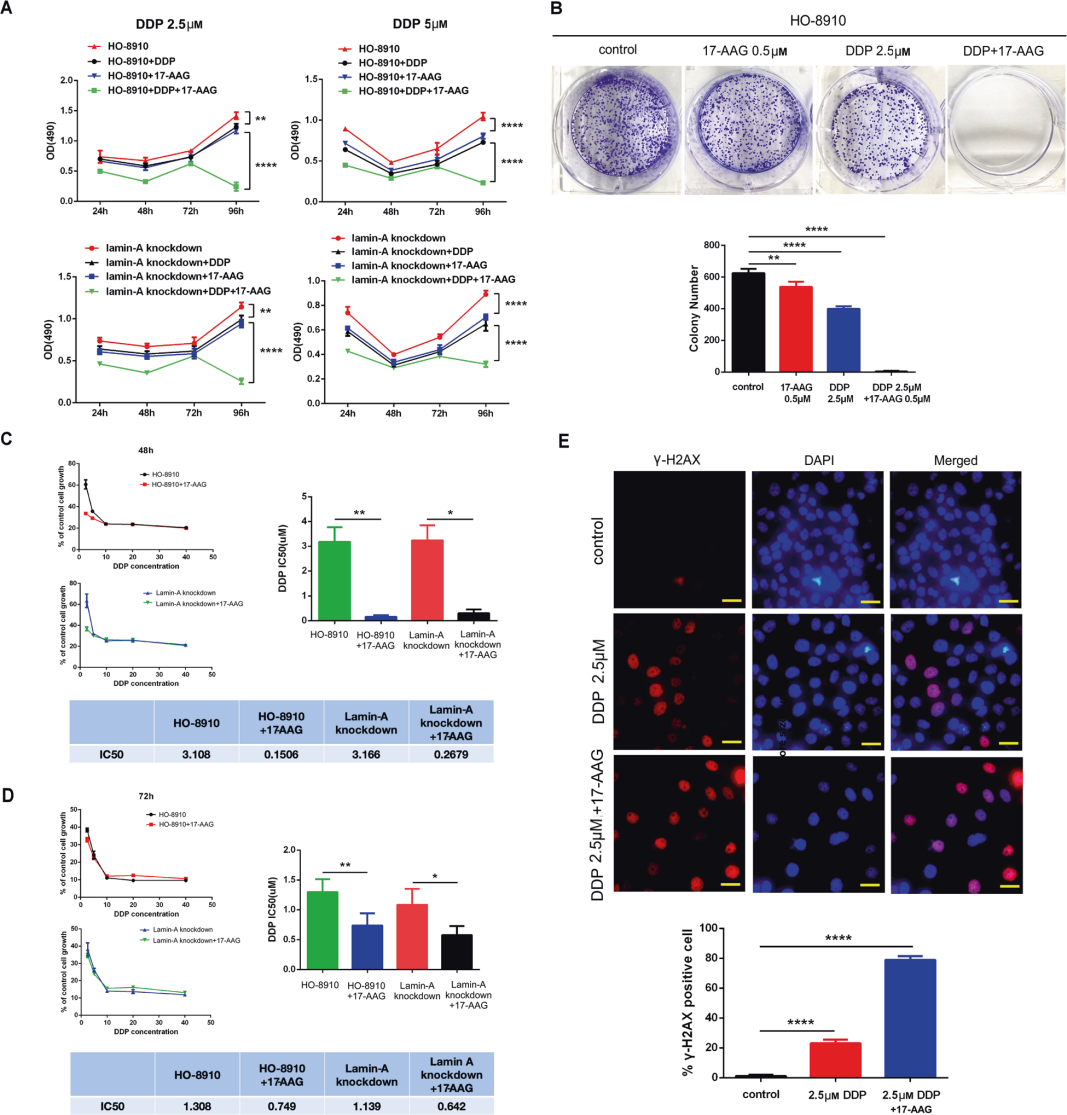
Combination of 17-AAG and DDP can efficiently inhibit the growth of ovarian cancer cells in vitro. **A** HO-8910 cells or stable lamin-A knockdown cells were treated with DDP (2.5 and 5 μM) or 17-AAG (0.5 μM) for 96 h, and cell viability was analyzed by CCK-8 assay. **B** Clonogenic assay: representative images of plates and colony numbers of different groups. Colonies containing more than 50 cells were counted. **C** and **D** Analysis and quantifications of IC50 value in HO-8910 cells (or stable lamin-A knockdown cells) treated with different concentrations of DDP (or combined with 17-AAG) for 48 h **C** and 72 h **D**. **E** Representative images of γ-H2AX foci and quantitative results of γ-H2AX positive cells. Scale bars, 50 μm. All the experiments were repeated three times. All the error bars indicated means ± SD. Statistical significance was concluded at *p < 0.05; **p < 0.01; ***p < 0.001.

Furthermore, the effect of Hsp90 inhibitor (17-AAG) on the sensitivity of ovarian cancer cells to a chemotherapeutic agent was investigated. To assess the inhibitory effect of 17-AAG and DDP on cell proliferation, CCK-8 assay was conducted. Compared with the control group, the proliferation ability of cells treated with 17-AAG (0.5 μM) alone decreased slightly, which was similar to (2.5 μM DDP group) or slightly higher (5 μM DDP group) than those treated with DDP alone (Fig. 5A). By comparison, cell viability markedly decreased when 17-AAG and DDP were combined, indicating that 17-AAG could enhance the toxicity of DDP to tumor cells, exhibiting a synergistic effect. Similar results were observed in SKOV-3, SKOV-3ip, and HO-8910 cells with stable lamin-A knockdown (Fig. 5A, supplementary Figure 1). In cell proliferation and drug resistance experiments, HO-8910 cells with stable lamin-A knockdown were used rather than cells with transient lamin-A knockdown, because transient transfection could not maintain enough time. The stable knockdown cells showed moderate knockdown of lamin-A verified by Western blotting and qRT-PCR (supplementary Figure 2).

Clonogenic assays are regarded as “gold standard” for measuring cellular sensitivity to drug treatment. Due to the slow growth of the surviving cells, the colony number significantly decreased when 17-AAG and DDP were combined, compared with DDP treated group and 17-AAG treated group, suggesting that the cell proliferation ability of HO-8910 was inhibited efficiently (Fig. 5B). Further verification was carried out in SKOV-3 and SKOV-3ip cells and got similar results (supplementary Figure 1).

Then the IC50 of cells in each group were calculated. In HO-8910 cells, 17-AAG treatment reduced the IC50 of DDP from 3.108 to 0.1506 after 48 h, respectively (from 1.308 to 0.749 after 72 h). Similarly, the IC50 value of DDP decreased from 3.166 to 0.2679 after 17-AAG treatment in the stable lamin-A knockdown group (from 1.139 to 0.642 after 72 h), and the difference was statistically significant (Figs. 5C, D). In addition, 17-AAG also led to an obvious decrease in the IC50 value of DDP in both SKOV-3 (from 40.53 to 7.125 after 48 h; from 24.46 to 1.655 after 72 h) and SKOV-3ip cells (from 60.57 to 30.50 after 48 h; from 30.67 to 6.288 after 72 h), suggesting that a lower dose of DDP could effectively inhibit the growth of tumor (Supplementary Figure 1).

Then, immunofluorescence was used to detect DNA damage in cells. As shown in Fig. 5E, the expression of γ-H2AX (a DNA double-strand break marker) was observed in the DDP group and 17-AAG combined with the DDP group, but the positive rate of the latter was higher, indicating more serious DNA damage after Hsp90 inhibition. Similar results were observed in stable lamin-A knockdown cells (Supplementary Figure 3). The positive rates of γ-H2AX in lamin-A knockdown cells were slightly higher than those of HO-8910 cells. Furthermore, Hsp90 overexpression could reduce the positive rate in the group treated with 17-AAG and DDP, suggesting that overexpression of Hsp90 in lamin-A deficient cells could restore and reverse the phenotype of increased DNA damage in combination with DPP to a certain extent.

## Combination of 17-AAG and DDP can significantly inhibit tumor growth of ovarian cancer in vivo and prolong the survival time of mice

**Fig. 6:**
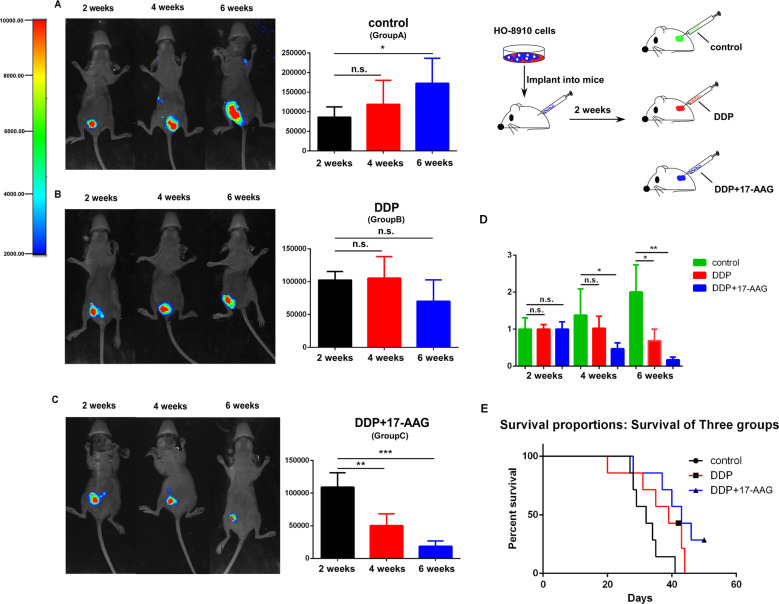
Combination of 17-AAG and DDP can significantly inhibit tumor growth of ovarian cancer in vivo and prolong the survival time of mice. **A**, **B,** and **C**: Representative images of animals and quantitative results of luciferase activity at different time points after inoculation with HO-8910 cells (A: group injected with normal saline as control, **B** group injected with DDP, **C** group injected with DDP + 17-AAG). Pictures were taken using an IVIS imaging system with i.p. injection of 100 mg/kg of D-luciferin. Color bars represented tumor cell intensity from low (blue) to high (red). **D** Analysis and quantifications of luciferase activities in all three groups, which were measured every two weeks. **E** Survival curves of nude mice. All the error bars indicated means ± SD. Statistical significance was concluded at *p < 0.05; **p < 0.01; ***p < 0.001.

Then the therapeutic potential of 17-AAG was investigated using nude mice intraperitoneal xenograft. Twelve mice were randomly divided into three groups. HO-8910 cells stably expressing luciferase were injected intraperitoneally (i.p.) into nude mice in each group. The luciferase activities were detected regularly to assess peritoneal tumor progression. Two weeks after injection, tumor formation was observed in all groups with no statistically significant. Then group B was intraperitoneally injected with DDP (3 mg/kg), group C was injected with DDP and 17-AAG (50 mg/kg), group A was injected with the same amount of normal saline as control. Two weeks later, the luciferase activity level (reflecting tumor volume in the peritoneal cavity) was slightly higher in group A, and there was no significant change of the luciferase activity level in group B, while the level decreased in group C (Fig.6A–C, Supplementary Figure 4). In addition, after 6 weeks,17-AAG-treated group showed obviously reduced tumor growth compared with groups A and B (*p* = 0.017, *p* = 0.003). Results showed that the luciferase activity level slightly decreased in group B with no statistically significance, while the level was even higher in group A (Fig. 6A–D, Supplementary Figure 4), indicating that a combination of 17-AAG and DDP could inhibit tumor growth more efficiently, which was in agreement with our in vitro studies. Besides, the survival time of group C was obviously longer than that of groups A and B, as shown in Fig. 6E, further supporting the notion that 17-AAG combined with DDP could prolong the survival time of mice.

## Discussion

Lamin-A is encoded by LMNA and plays an important role in many biological processes of cells. The previous studies have found that lamin-A is related to DSBs repair [13], which is the most serious type of DNA damage, leading to genomic instability, chromosome damage, tumorigenesis, and cell death [9, 32]. The underlying relationship between the lamin-A and DNA damage repair proteins is poorly understood, and we speculate that lamin-A can affect DNA damage repair through its interacting proteins. Deriving from human embryonic kidney cells, 293 T cells do not express lamin-A and are easy to be transfected, so flag-lamin-A was transfected into 293 T cells (Fig. 1A). Then, a series of proteins that may interact with lamin-A were obtained (Fig. 1B-D). Among them, Hsp90 was found to be related to DNA damage repair. Hsp90 is a molecular chaperone, which is closely related to the folding, stability, and activation of proteins [33]. Cancer cells widely rely on Hsp90 to assist signal transduction, making Hsp90 a potential target for cancer therapy [34].

Hsp90 can maintain homeostasis in cells, and part of its function is to promote or participate in many protein–protein interactions [35]. In our research, the interaction between lamin-A and Hsp90 was confirmed. Furthermore, plasmids expressing different lamin-A derivatives were co-transfected with his-Hsp90 into 293 T cells. Results showed that lamin-A could interact with Hsp90 via the domain of lamin-A^1-430^ (Fig. 2).

To further study the relationship between them, lamin-A was knockdown in ovarian cancer cells, but Hsp90 did not change significantly (Fig. 3B). However, immunofluorescence suggested that Hsp90 decreased in the cytoplasm (Fig. 3C) and separation of nucleoplasmic proteins further verified the above results (Figs. 3E, 3F). In addition, we noticed that in the further knockdown lamin-A group, lamin-A and Hsp90 were predominantly co-localized around the nuclear membrane (Fig. 3D). We speculate that lamin-A knockdown could promote the nuclear translocation of Hsp90, thus affecting DNA damage proteins and causing DNA repair defects. It is well known that Hsp90 mainly locates in the cytoplasm but it can also be transported to other parts of the cell, such as the extracellular matrix, where it can regulate the invasion of tumor cells [36]. Research also showed that Hsp90 could be transferred to the nucleus [37, 38]. Hsp90 does not have NLS, which is necessary for nuclear localization. Therefore, its nuclear transport may occur through co-transportation with chaperone proteins. According to our research, the interaction between lamin-A and Hsp90 may be related to the nucleocytoplasmic transport of Hsp90. Hsp90 could maintain its balance and activity in cells by interacting with lamin-A. Moderate knockdown of lamin-A had little effect on the distribution of Hsp90 but when lamin-A was a further knockdown, Hsp90 could not be effectively transported and gathered near the nuclear membrane, which affecting its activity and DNA damage. However, the mechanism of how lamin-A regulates the nuclear transport of Hsp90 through interaction is under investigation.

So far, the function of nuclear Hsp90 is still unclear. Researches showed that nuclear Hsp90 could bind to MRN complex and maintain its activity [39, 40], others found that Hsp90 could interact with DDR proteins [26] and regulate the recruitment of 53BP1 to DSBs [41], suggesting Hsp90 may be related to DNA damage repair but the specific mechanism remains to be studied. Rad50 and Ku80 are key proteins for DSBs repair [42], which were downregulated after lamin-A further knockdown in our previous research [13]. Here, the effect of Hsp90 inhibition on DSBs repair was further studied. Results showed that expression of DSBs proteins decreased after inhibition of Hsp90 (Fig. 4), suggesting impairment of both NHEJ and HR pathways in DNA damage repair, which was similar to lamin-A deficiency in our previous research. Besides, other researchers also showed that multiple components of the DSBs repair machinery, including BRCA1, BRCA2, DNA-PKcs, and the MRE11/RAD50/NBN complex, were described to be client proteins of Hsp90 [26, 28, 43], and our study further proved that Hsp90 was closely related to DNA damage repair, making it act as a regulator of diverse DDR pathways.

The response of cells to DNA damage is an important determinant of tumor development. Chemoradiotherapy usually induces DSBs to exert cytotoxicity, so alterations in DDR can drive sensitivity or resistance to these agents [44]. Inhibition of DNA damage repair proteins can not only be used as a sensitizer combined with DNA damaging agent but also as a single antitumor drug [45]. For example, BRCA1 deficient cells had DNA repair defects and increased sensitivity to PARP inhibitors [46–48]. Besides, Hsp90 inhibitor could be a radiosensitizer by inhibiting Rad51 mediated HR repair pathway [49]. It was also found that Hsp90 inhibitors could affect DNA damage after ionizing radiation but the reason was unclear [50].

To test the effects of 17-AAG on cell chemosensitivity, we assessed cell survival and found that cell proliferation ability decreased significantly when 17-AAG and DDP were combined (Figs. 5A, 5B, supplementary Figure 1). Furthermore, 17-AAG led to an obvious decrease in the IC50 value of DDP in ovarian cancer cell lines (Figs. 5C, 5D, supplementary Figure 1). Results indicated that 17-AAG could enhance the toxicity of DDP to tumor cells, exhibiting a synergistic effect. This is of great value for the application of antitumor drugs, delaying tumor progression, and improving survival. Moreover, we explored the underlying mechanisms. Immunofluorescence showed that the positive rate of γ-H2AX increased in group treated with 17-AAG and DDP, suggesting that DNA damage could not be repaired in time, which may be due to impairment of both NHEJ and HR repair pathways. Furthermore, cells with stable lamin-A knockdown exhibited more serious DNA damage compared with HO-8910 cells (Supplementary Figure 3), and overexpression of Hsp90 could reduce the positive rate of γ-H2AX to a certain extent in 17-AAG combined with the DDP group, confirming that Hsp90 overexpression in lamin-A deficient cells could reverse the phenotype of increased DNA damage in combination with DPP.

In addition, the therapeutic potential of 17-AAG in ovarian cancer was examined in vivo. We investigated its capacity to suppress tumor growth using a nude mice xenograft model. The 17-AAG treated group showed significantly reduced the tumor growth compared with the other groups (Fig. 6A–D, Supplementary Figure 4), which was in agreement with our in vitro studies. Besides, the survival time of mice was significantly longer (Fig. 6E), further supporting the notion that the combination of 17-AAG and DDP could enhance the antitumor effect of DDP, so as to better inhibit the growth of the tumor, prolong the survival period and improve the prognosis, which may have a certain guiding significance for clinical medication.

## Supplementary information

Supplementary Figure Legends

Supplementary Figure 1

Supplementary Figure 2

Supplementary Figure 3

Supplementary Figure 4

## Data Availability

All data generated or analyzed during this study are included in this published article and its supplementary information files.
