# Supplementary material for: Lamin-A interacting protein Hsp90 is required for DNA damage repair and chemoresistance of ovarian cancer cells
Source: Cell Death Dis. 2021 Aug 12;12(8):786. doi: 10.1038/s41419-021-04074-z (PMC8358027; doi:10.1038/s41419-021-04074-z)
Supplement: Supplementary file 1 — Supplementary Figure Legends [file 41419_2021_4074_MOESM1_ESM.docx]

**Supplementary Figure 1.** A: Clonogenic assay: Representative images of plates and colony numbers of different groups in SKOV-3 and SKOV-3ip cells. Colonies containing more than 50 cells were counted. B: SKOV-3 or SKOV-3ip cells were treated with DDP (2.5μM, 5μM) or 17-AAG (0.5μM) for 96 h, and cell viability was analyzed by CCK-8 assay. (C and D): Analysis and quantifications of IC50 value in SKOV-3 and SKOV-3ip cells treated with different concentrations of DDP (or combined with 17-AAG) for 48h (C) and 72h (D). All the experiments were repeated three times. All the error bars indicated means ± SD. Statistical significance was concluded at **p*<0.05; ***p*<0.01; ****p*<0.001.

**Supplementary Figure 2.** Knockdown efficiency of stable lamin-A knockdown cells was determined by Western blotting (A) and qRT-PCR (B). All the error bars indicated means ± SD. Statistical significance was concluded at **p*<0.05; ***p*<0.01; ****p*<0.001.

**Supplementary Figure 3.** A: Representative images of γ-H2AX foci in groups of stable lamin-A knockdown cells. Scale bars, 50μm. B: Representative images of γ-H2AX foci after Hsp90 overexpression in groups of stable lamin-A knockdown cells. Scale bars, 50μm. C: Quantitative results of γ-H2AX positive cells. All the error bars indicated means ± SD. Statistical significance was concluded at **p*<0.05; ***p*<0.01; ****p*<0.001.

**Supplementary Figure 4.** (A, B and C): Representative images illustrating the tumor growth of the other nude mice at different time points (A: group injected with normal saline as control, B: group injected with DDP, C: group injected with DDP+17AAG). Pictures were taken using an IVIS imaging system with i.p. injection of 100 mg/kg of D-luciferin. Color bars represented tumor cell intensity from low (blue) to high (red).
