# Supplementary figures and images for: Lamin-A interacting protein Hsp90 is required for DNA damage repair and chemoresistance of ovarian cancer cells
Source: Cell Death Dis. 2021 Aug 12;12(8):786. doi: 10.1038/s41419-021-04074-z (PMC8358027; doi:10.1038/s41419-021-04074-z)

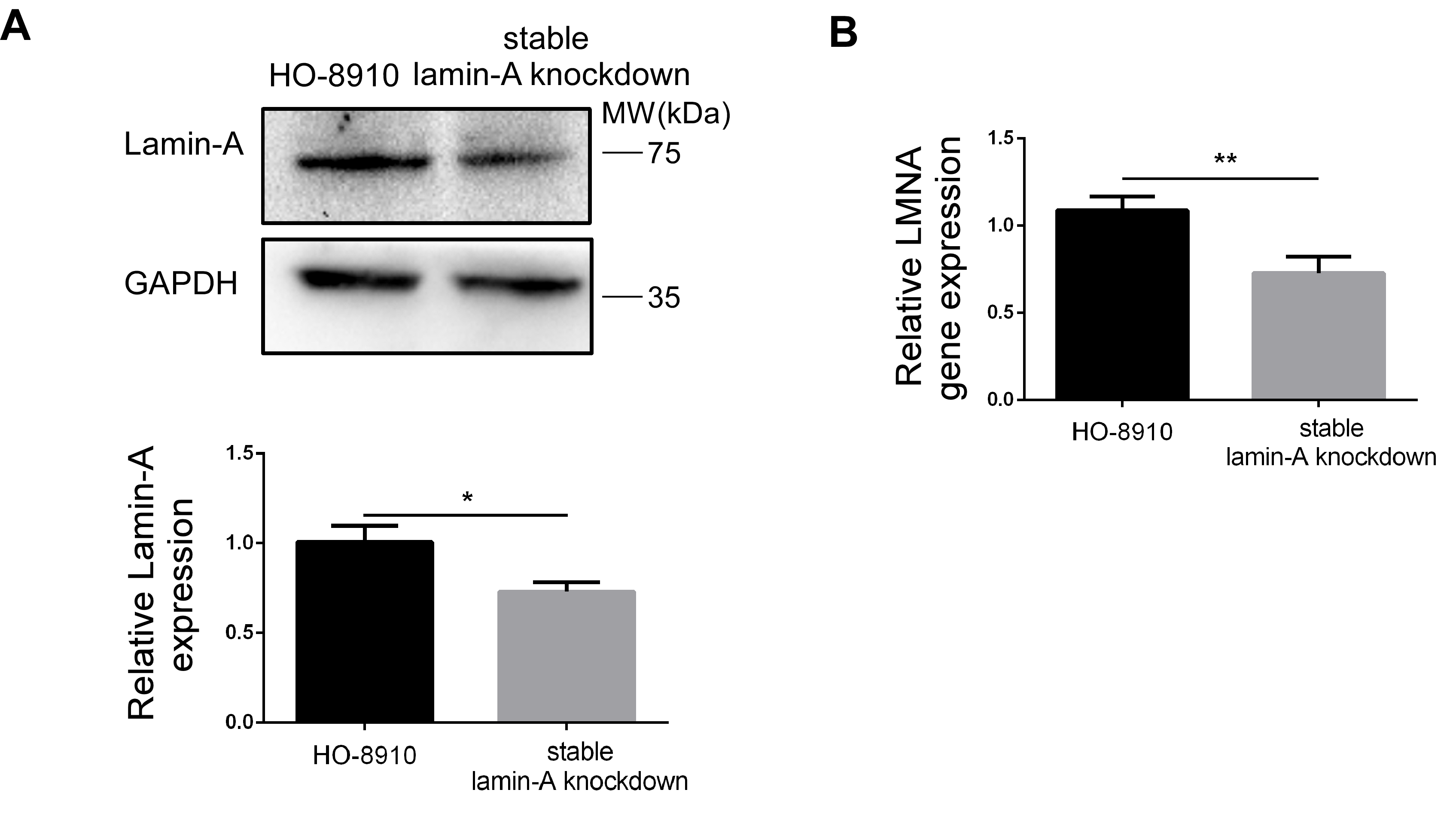

Supplement: Supplementary file 3 — Supplementary Figure 2 [file 41419_2021_4074_MOESM3_ESM.tif]
